# Quantum correlations beyond entanglement in a classical-channel model of gravity

**DOI:** 10.1038/s41598-022-22212-1

**Published:** 2022-10-21

**Authors:** Federico Roccati, Benedetto Militello, Emilio Fiordilino, Rosario Iaria, Luciano Burderi, Tiziana Di Salvo, Francesco Ciccarello

**Affiliations:** 1 Department of Physics and Materials Science, University of Luxembourg, L-1511 Luxembourg, Luxembourg; 2 Dipartimento di Fisica e Chimica – Emilio Segrè, Università degli Studi di Palermo, via Archirafi 36, 90123 Palermo, Italy; 3 INFN Sezione di Catania, via Santa Sofia 64, 95123 Catania, Italy; 4 Dipartimento di Fisica, Università degli Studi di Cagliari, SP Monserrato-Sestu, KM 0.7, 09042 Monserrato, Italy; 5 NEST, Istituto Nanoscienze-CNR, Piazza S. Silvestro 12, 56127 Pisa, Italy

**Keywords:** Quantum information, Quantum mechanics

## Abstract

A direct quantization of the Newtonian interaction between two masses is known to establish entanglement, which if detected would witness the quantum nature of the gravitational field. Gravitational interaction is yet compatible also with gravitational decoherence models relying on classical channels, hence unable to create entanglement. Here, we show in paradigmatic cases that, despite the absence of entanglement, a classical-channel model of gravity can still establish quantum correlations in the form of quantum discord between two masses. This is demonstrated for the Kafri–Taylor–Milburn (KTM) model and a recently proposed dissipative extension of this. In both cases, starting from an uncorrelated state, a significant amount of discord is generally created. This eventually decays in the KTM model, while it converges to a small stationary value in its dissipative extension. We also find that initial local squeezing on the state of the masses can significanlty enhance the generated discord.

## Introduction

Among the four fundamental forces, gravity is the only one whose quantum nature was never demonstrated. Currently, there is growing confidence that the hindrances to testing the quantumness of the gravitional field due to Planck-scale limits could be overcome through table-top experiments^1^. Some of these, in particular, propose that detecting entanglement between two masses would witness the quantum nature of the gravitational field mediating their mutual interaction^2–4^.

To date, as no phenomena are yet known where quantum mechanics and gravity coexist, the possibility that gravity could be just classical cannot be ruled out. If so, however, as the quantum nature of matter is well-established one should yet conceive a hybrid scenario where a classical channel mediates gravitational interactions between intrinsically quantum masses. Several models of this kind have been proposed^5–10^. In these models, typically, the conjectured classical channel gives rise to the Newtonian potential, yet causing at the same time decoherence affecting the quantum masses^11^. Decoherence plays against the entanglement that would arise from the Newtonian potential, the net result being that no entanglement can be generated in such models in agreement with their classical nature^5–10^. More specifically, this follows from the fact that they rely only on local operations and classical communication (LOCC), namely operations unable to create entanglement (this being a distinctive property of entanglement itself)^12^.

Notwithstanding the above, classical channel models of gravity could be still compatible with establishment of quantum correlations (QCs). Indeed, entanglement is not the most general form in which correlations of a non-classical nature can manifest. This has been known since the early 2000s, when it was introduced a general quantifier of QCs usually going under the name of *quantum discord* or simply “discord”^13,14^. Notably, while any entangled state has non-vanishing discord, the converse does not hold: there are states which—although fully separable (non-entangled)—still feature non-local correlations incompatible with classical physics. Remarkably, these zero-entanglement QCs can be harnessed as a resource for a number of quantum information processing tasks, which was confirmed in a number of experiments^15,16^, including the possibility to generate^17–20^ and distribute^21–24^ entanglement. Remarkably, unlike entanglement, discord *can* be created through LOCC. For instance, a local dissipative channel can turn a classically-correlated state into one with non-zero discord (but still disentangled)^25,26^.

With the above motivations, this work addresses the question as to whether or not quantum correlations according to this extended paradigm can be generated in a classical channel model of gravity and, if so, whether they are stable or eventually decay at large times. We carry out this task in the case study of the Kafri Taylor Milburn (KTM) model^6^ and its recently proposed dissipative version^10^. This allows to consider a relatively simple system made out of a pair of quantum harmonic oscillators for which effective techniques were developed to compute quantum discord^27,28^ (calculation of discord is generally quite challenging^15,16,29^). We will in particular show that, although entanglement never shows up, starting from a fully uncorrelated state QCs are indeed created during the dynamics in a significant amount (compared to the total correlations). Such generated discord eventually undergoes a slow decay in the case of the KTM model, while it converges to a finite, although small, stationary value for its dissipative extension^10^.

## Results

### Quantized Newtonian potential

**Figure 1:**
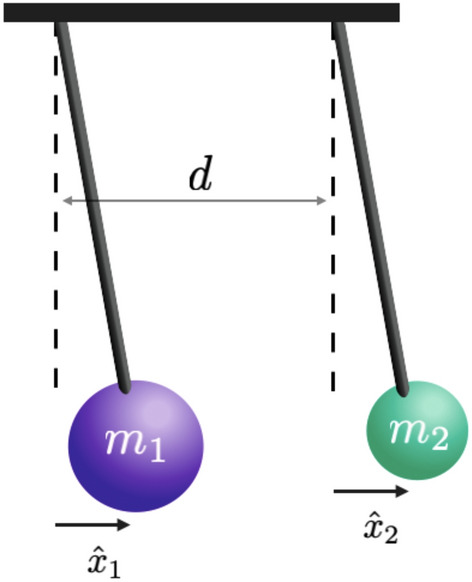
System: two suspended masses (pendula) $m_1$ and $m_2$, where $x_j$ is the displacement of the *j*th mass from the respective equlibrium position and *d* the distance between the equilibrium positions.

Consider two suspended masses $m_1$ and $m_2$ (see Fig. 1) subject to mutual Newtonian attraction. In the usual regime of small oscillations, the masses are effectively modeled as a pair of independent quantum harmonic oscillators so that the Hamiltonian reads

1 $$\begin{aligned} {\hat{H}}={\hat{H}}_0-G\,\frac{m_1m_2}{\sqrt{{\hat{x}}_{21}^2}}\,, \end{aligned}$$

with ${\hat{H}}_0$ the free Hamiltonian given by

2 $$\begin{aligned} {\hat{H}}_0 = \sum _{j=1}^2 \left( \frac{{\hat{p}}_j^2}{2m_j} + \frac{1}{2} m_j\omega _j^2\,{\hat{x}}_j^2\right) \, \end{aligned}$$

where *G* is the gravitational constant while ${\hat{x}}_{21}={\hat{x}}_2-{\hat{x}}_1$.

In the standard regime of small displacements from the equilibrium positions and redefining ${\hat{x}}_j$ as the displacement of mass $m_j$ from the equilibrium position (i.e., the one for $G\ne 0$), Hamiltonian (1) is well-approximated by

3 $$\begin{aligned} {\hat{H}}= {\hat{H}}'_0+ {\hat{V}} \end{aligned}$$

where ${\hat{H}}'_0$ is obtained from ${\hat{H}}_0$ [cf. Eq. (2)] by replacing $\omega _j^2$ with $\omega _j^2 - K/m_j$, while ${\hat{V}}$ embodies the (linearized) Netwon’s interaction Hamiltonian

4 $$\begin{aligned} {\hat{V}}=K\,{\hat{x}}_1{\hat{x}}_2\, \end{aligned}$$

with

5 $$\begin{aligned} K=2G\, \frac{m_1m_2}{d^3}\,, \end{aligned}$$

where *d* is the distance between the equilibrium positions of the two masses [Eq. (3) is obtained by expanding the Newtonian potential to the 2nd order in the mass displacements as ${\hat{V}}\simeq -G\,\frac{m_1m_2}{d} \left( 1+\frac{{\hat{x}}_{21}}{d}-\frac{{\hat{x}}_{21}^2}{d^2}\right)$ and eliminating next the linear terms through the aforementioned redefinition of each mass coordinate].

Potential (4) results from the mere quantization of the *static* Newtonian potential irrespective of what channel or charge carriers mediate the gravitational attraction. In this model, the two masses jointly embody a closed system (no decoherence). Accordingly, their dynamics is unitary and thus governed by the Liouville-von Neumann equation (equivalent to the Schrödinger equation)

6 $$\begin{aligned} {\dot{\rho }} = -i[{\hat{H}} ,\rho ] \end{aligned}$$

with $\rho$ the joint density operator of the two masses and ${\hat{H}}$ given by (3). Here and throughout the paper we set $\hbar =1$.

As generally expected for quantum systems subject to a direct mutual interaction and isolated from an external environment, the Newtonian potential ${\hat{V}}$ causes establishment of entanglement between the two masses when these start in a fully uncorrelated state, which was shown in Ref.^4^.

### Creation of quantum correlations in the KTM model

#### KTM model

The KTM model^6^ assumes a specific, fully *classical*, channel mediating the gravitational interaction. This gives rise to the Newtonian potential ${\hat{V}}$ which is however accompanied by additional decoherence affecting the two-mass system. Specifically, the classical channel consists of local measurements plus feedback as briefly sketched next (see e.g. Ref.^30^ for a more detailed description). The position of $m_1$ is first instantaneously measured, the outcome being $x_1$. A local field described by a Hamiltonian term $\propto \!x_1 {\hat{x}}_2$ is then applied on mass 2 for an infinitesimal time (note that here only ${\hat{x}}_2$ is an operator). Analogous operations with swapped roles of systems 1 and 2 are next carried out and the whole process iterated over and over during the entire time evolution. Note that such a dynamics is necessarily stochastic since measurements in quantum mechanics are probabilistic. The average (so called “unconditional”) dynamics, however, is deterministic and demonstrably described by the master equation (ME)^6^

7 $$\begin{aligned} {\dot{\rho }} = -i[{\hat{H}},\rho ] +\sum _{j=1}^2 \left( \lambda + \frac{K^2}{4\lambda }\right) {\mathcal {D}}[{\hat{x}}_j]\rho \end{aligned}$$

with ${\hat{H}}$ and *K* the same as (3) and (5), respectively, and where we set ${\mathcal {D}}[{\hat{O}}]\rho ={\hat{O}}\rho \,{\hat{O}}^\dagger -\frac{1}{2}({\hat{O}}^\dagger {\hat{O}}\rho +\rho \,{\hat{O}}^\dagger {\hat{O}})$. Here, $\lambda$ measures the characteristic rate of the measurement-feedback operation [this rate is generally dependent on the susbsystem ($m_1$ or $m_2$). Here, we it assumed it to be independent of the subsystem for the sake of simplicity].

The terms containing $\lambda$ (jointly called dissipator) describe decoherence affecting the two masses, which makes the system effectively open and its dynamics non-unitary. Notably, decoherence is minimized for $\lambda =K/2$, a value which we will set throughout the remainder.

Without dissipator, ME (7) would reduce to (6), showing that the classical channel gives rise to the canonical gravitational attraction yet introducing at the same time ineliminable decoherence (observe that the dissipator is non-zero for any value of $\lambda$).

We point out that the dissipator in (7) is the sum of two *local* dissipators: such “local noise” (as is often referred to) is well-known to generally spoil entanglement. This counteracts the effect of the Hamiltonian term in ME (7) which instead can create entanglement due to the Newtonian potential (4) as discussed in the previous section. The net result is that the dynamics described by ME (7) is unable to create entanglement. Physically, this is due to the fully classical nature of the gravitational channel described above which relies solely on LOCC. In fact, therefore, the KTM model cannot generate entanglement by construction.

It is convenient to introduce rescaled positions and momenta as (recall that $\hbar =1$) ${\hat{X}}_j =\sqrt{m\omega }\,{\hat{x}}_j\,,\,\,\,{\hat{P}}_j = {\hat{p}}_j/\sqrt{m\omega }$. In terms of these dimensionless operators, master equation (7) for $\lambda =K/2$ (minimal decoherence) can be arranged as

8 $$\begin{aligned} {\dot{\rho }} = -i[{\hat{H}},\rho ] + \eta \omega \sum _{j=1}^2 {\mathcal {D}}[{\hat{X}}_j]\rho \end{aligned}$$

with

9 $$\begin{aligned} {\hat{H}}=\omega \left[ \frac{1}{2} \sum _{j=1}^2 \left( {\hat{P}}_j^2 + (1-\eta ){\hat{X}}_j^2 \right) + \eta {\hat{X}}_1{\hat{X}}_2 \right] \,. \end{aligned}$$

Here, in line with other works^31^ we introduced the dimensionless parameter

10 $$\begin{aligned} \eta =\frac{K}{m\omega ^2}\,, \end{aligned}$$

namely the ratio between the gravitational coupling strength and the characteristic energy of the harmonic confinement of each mass. Thus $\eta$ measures the effective strength of the gravitational interaction. Since such interaction is typically very weak, $\eta$ should always be considered such that $\eta \ll 1$. In the remainder, we will frequently take advantage of this condition to make approximations.

#### Equation of motion for the covariance matrix

We will consider throughout the two-mass system initially prepared in a *Gaussian state*, i.e. a state whose characteristic function is Gaussian^32^. Many relevant states—such as coherent, thermal and squeezed states—belong to this large class. The form of the KTM master equation (7) is such that if the system starts in a Gaussian state then its state will remain Gaussian at any time^32^.

By definition, a Gaussian state of the two masses (quantum harmonic oscillators) is fully specified by the first and second moments $\langle {\hat{O}}_m \rangle$ and $\langle {\hat{O}}_m {\hat{O}}_n \rangle$ with $m,n=1,2,3,4$, where $\langle {\hat{A}} \rangle =\mathrm{Tr}\{\rho {\hat{A}}\}$ and ${\hat{O}}_1={\hat{X}}_1$, ${\hat{O}}_2={\hat{P}}_1$, ${\hat{O}}_3={\hat{X}}_2$, ${\hat{O}}_4={\hat{P}}_2$. For our purpose of calculating correlations (as will become clear later) it is sufficient to consider the covariance matrix $\sigma$ whose entries are defined as $\sigma _{mn} = \langle {\hat{O}}_m {\hat{O}}_n+{\hat{O}}_n {\hat{O}}_m\rangle -2 \langle {\hat{O}}_m \rangle \langle {\hat{O}}_n \rangle$.

In the KTM model, master equation (7) entails that $\sigma$ evolves in time according to the Lyapunov equation of motion^33^

11 $$\begin{aligned} \dot{\sigma } = Y\sigma +\sigma \, Y^{\mathrm{T}} + 4 D \end{aligned}$$

with

12 $$\begin{aligned} Y = \left( \begin{array}{cccc} Y_{11} &{} \quad Y_{12} \\ Y_{12}&{} \quad Y_{11} \\ \end{array} \right) , \qquad \text {where}\,\,\,\,\,\,\, Y_{11} = \omega \left( \begin{array}{cccc} 0&{}1\\ \eta {-}1 &{} \quad 0 \end{array} \right) ,\,\,\,\,\, Y_{12} = \left( \begin{array}{cccc} 0 &{} \quad 0\\ -\eta \omega &{} \quad 0 \end{array} \right) , \end{aligned}$$

and with *D* the diagonal matrix defined by

13 $$\begin{aligned} D = \left( \begin{array}{cccc} D_{11} &{} \quad 0 \\ 0&{} \quad D_{11} \\ \end{array} \right) \qquad \text {where}\,\,\,\,\,\,\, D_{11} = \left( \begin{array}{cccc} 0&{} \quad 0\\ 0 &{} \quad \tfrac{\eta \omega }{2} \end{array} \right) , \end{aligned}$$

[note that all these matrices are symmetric under the exchange $1\leftrightarrow 2$ since so is master equation (8). The same property for the same quantities also holds in the dissipative KTM model and, additionally, for any covariance matrix appearing throughout].

The solution of the linear equation of motion (11) reads

14 $$\begin{aligned} \sigma (t) = e^{Yt}\sigma _0\, e^{Y^Tt} + 4\int _{0}^{t}\text {d}s\, e^{Ys}D\,e^{Y^Ts} \end{aligned}$$

with $\sigma _0$ the covariance matrix at $t=0$ (the explicit analytical expression is cumbersome and thus not reported here).

The knowledge of the covariance matrix at any time *t* is enough to compute correlations of any kind (quantum or not) between the two masses.

#### Dynamics of of quantum correlations

Our aim is assessing the ability of the classical channel in the KTM model to create QCs and, if any, analyzing their temporal behaviour. Accordingly, we naturally focus throughout on initial product states, i.e. of the form

15 $$\begin{aligned} \rho _0=\varrho _1\otimes \varrho _2 \end{aligned}$$

with $\varrho _j$ the initial state of mass *j*. State (15) features no correlations of any kind between the two masses (thus mutual information ${{\mathscr {I}}}$ and of course ${{\mathscr {D}}}$ vanish).

We will next consider two different types of initial states: coherent and squeezed.

When $\varrho _j$ is a *coherent state* it can be expressed as $\varrho _j=|\alpha _j\rangle \!\langle \alpha _j|$ with $|\alpha _j\rangle =e^{\alpha _j {\hat{a}}_j^\dag -\alpha _j^*{\hat{a}}_j}|0\rangle _j$, where $|0\rangle _j$ the vacuum state and ${\hat{a}}_j$ the usual annihilation operator. In this case, the covariance matrix corresponding to (15) is simply given by

16 $$\begin{aligned} \sigma _0=\text {diag}(1,1,1,1)\,, \end{aligned}$$

which is independent of the coherent-state amplitude $\alpha _j$.

**Figure 2:**
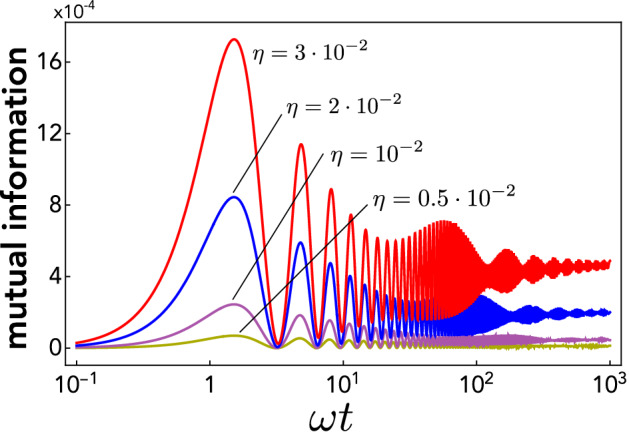
Semi-log plot of mutual information ${{\mathscr {I}}}$ against time (in units of $\omega ^{-1}$) when each mass starts in a coherent state. Note that the growing frequency of damped oscillation is due to the logarithmic scale.

To benchmark the dynamics and amount of quantum correlations to be analysed shortly, we first study the behaviour of mutual information ${{\mathscr {I}}}$ (measuring total correlations, either classical or quantum), which can be calculated straightforwardly from Eqs. (24) and (16). The behaviour of ${{\mathscr {I}}}$ against time (rescaled in units of $\omega ^{-1}$) is reported in Fig. 2 for growing values of the effective gravitational interaction strength $\eta$ [recall definition (10)].

We see that ${{\mathscr {I}}}$ first grows monotonically from zero until reaching its maximum value, then undergoes damped oscillations (exhibiting vanishing minima) and eventually asymptotically converges to a finite stationary value. Note that, as is reasonable, the maximum and asymptotic values grow with the effective gravitational interaction strength $\eta$.

Let us now address quantum correlations in the form of quantum discord. Being defined as the mismatch between mutual information and classical correlations^13–15^, it captures the pure quantum contribution to correlations in a bipartite system. This discrepancy can only appear in the quantum realm as classical correlations rely upon the knowledge of the state of a subsystem (local measurement). For the relevant case of Gaussian states, measurements can be restricted to Gaussian ones^34^, yielding a closed analytical expression of the so called Gaussian discord^27,28^ (see “Methods” section for more details).

**Figure 3:**
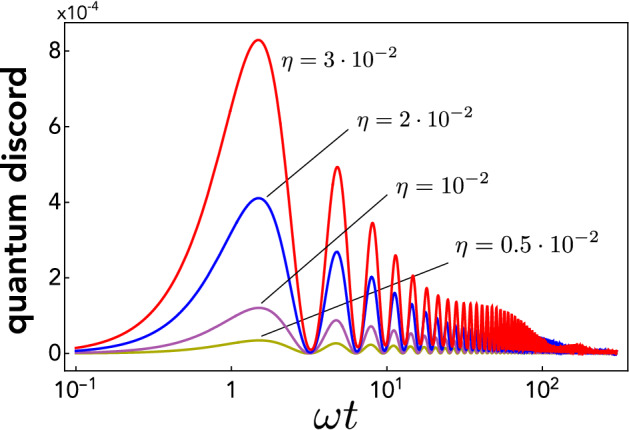
Semi-log plot of quantum discord ${{\mathscr {D}}}$ against time (in units of $\omega ^{-1}$) when each mass starts in a coherent state. Discord ${{\mathscr {D}}}$ was calculated using Eq. (27).

The dynamics of quantum correlations is computed by plugging $\sigma _0$ into (14) and next working out the corresponding evolved Gaussian discord ${{\mathscr {D}}}(t)$, which yields an exact although cumbersome expression (not reported here). The time behaviour of ${{\mathscr {D}}}$ is shown in Fig. 3 for increasing coupling strengths $\eta$. Discord first grows monotonically until reaching a maximum and then undergoes damped oscillations with vanishing minima. The generation of quantum correlations can be justified noting that the dissipator in Eq. (7) can be written as ${\mathcal {D}}[{\hat{x}}]\rho \propto {\mathcal {D}}[{\hat{a}}]\rho + {\mathcal {D}}[{\hat{a}} ^\dagger ]\rho + F({\hat{a}},{\hat{a}}^\dagger ; \rho )$, where $F({\hat{a}},{\hat{a}}^\dagger ; \rho )$ contains all the remaining terms. While the jump operator ${\hat{a}}$ acts trivially on a coherent state, the operator ${\hat{a}}^\dagger$ turns it into a mixture of non-orthogonal (coherent) states, a key ingredient to get non-zero discord^35^.

Thereby, quantum correlations indeed show up, thus confirming that they can be created by the classical gravitational channel. The amount of quantum correlations established in the transient is significant, which can be seen e.g. by noting that the first maximum of ${{\mathscr {D}}}$ is about half as that of mutual information [see Fig. 2]. Overall, the temporal dynamics of discord is similar to mutual information except at long times. Indeed, the plots indicate that, within our numerical capabilities, quantum correlations are unstable, dropping off with a long tail for $t\rightarrow \infty$. A rigorous proof that QCs vanish in the long-time limit is demanding due to the cumbersome expression of ${{\mathscr {D}}}(t)$ and the very long tail. Remarkably, however, this decay to zero can be shown analytically in the realistic regime of small $\eta$ (due to the weakness of gravitational interaction). Indeed, in this case, the long-time expression of discord can be worked out as

17 $$\begin{aligned} \mathscr {D}(\tau ) \simeq \frac{1}{2} \left[ (1+\eta \tau ) \log \frac{\eta \tau }{2+\eta \tau } + \log \left( 1-(\eta \tau )^{-4}\right) - \frac{(\eta \tau )^2}{1+\eta \tau } \log \frac{(\eta \tau )^2-\eta \tau -1}{(\eta \tau )^2+\eta \tau +1} \right] \,\,\,\,\, \end{aligned}$$

with $\tau =\omega t$. This is easily shown to vanish in the limit $\tau \rightarrow \infty$.

To sum up, the above shows that quantum correlations are indeed created by the gravitational interaction in the transient and in a significant amount, but eventually (although slowly) fade away.

**Figure 4:**
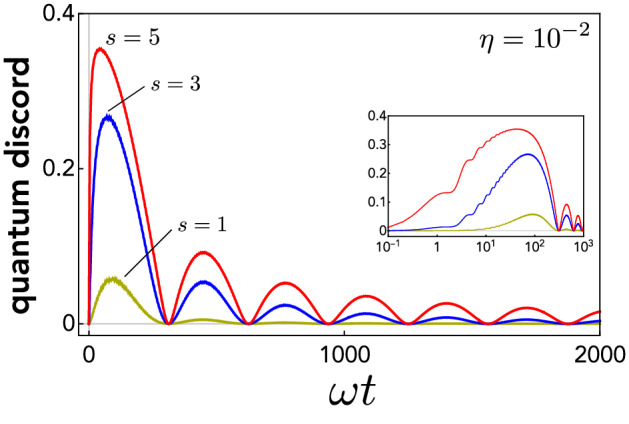
Lin-lin plot of quantum discord against time (in units of $\omega ^{-1}$) when each mass starts in a squeezed state $|s_j\rangle$ for different values of the squeezing parameter *s*. We set $\eta =10^{-2}$. The inset contains the lin-log version of the plot. Discord ${{\mathscr {D}}}$ was calculated using Eq. (27).

Coherent states are the most classical states in that both the field quadratures ${\hat{x}}_i$ and ${\hat{p}}_j$ have minimum uncertainty. In contrast, we next take each $\varrho _j$ [cf. Eq. (15)] to be a *squeezed state*^36^ where the uncertainty on one of the two quadratures is “squeezed” (and the other one is consequently enhanced as imposed by the uncertaintly principle) [the non-classicality mentioned here lies in the reduced state of each oscillator (at $t=0$) and not in the correlations between the two oscillators, recall that initial state (15) is fully uncorrelated].

A squeezed state of the *j*th mass reads $\eta _j=|s_j\rangle \!\langle s_j|$ with $|s_j\rangle =e^{[s_j ({\hat{a}}_j^2)^\dag -s_j^*{\hat{a}}_j^2]/2}|0_j\rangle$, where $s_j$ is the squeezing parameter. For simplicity, we assume the same squeezing on both the oscillators, i.e., $s_1=s_2=s$. Accordingly, the covariance matrix corresponding to the initial state (15) in this case can be worked out as

18 $$\begin{aligned} \sigma _0 = \left( \begin{array}{cc} S_2 &{} \quad 0 \\ 0 &{} \quad S_2 \\ \end{array} \right) \, \end{aligned}$$

with

19 $$\begin{aligned} S_2 = \left( \begin{array}{cc} \cosh s + \sinh s &{} \quad 0 \\ 0 &{} \quad \cosh s - \sinh s \\ \end{array} \right) . \end{aligned}$$

For $s=0$ (no squeezing) $\sigma _0$ coincides with (16) as in this limit the initial state of each mass reduces to the vacuum state (which can be seen as a zero-amplitude coherent state).

The calculation of discord (25) corresponding to (18) yields again an involved expression, which is hard to arrange in a form as compact as Eq. (17) even restricting to specific regimes.

In Fig. 4, we set the representative value of coupling strength $\eta =10^{-2}$ and study the effect of squeezing (as measured by *s*) on generation of discord. A dynamics qualitatively similar to the case of coherent states [cf. Fig. 3] occurs, which features an initial growth towards a maximum followed by damped oscillations characterized by a slow decay time. A remarkable difference from coherent states [cf. Fig. 3] yet stands out in that discord can reach far higher values during the transient, even orders of magnitudes larger (but still below the entanglement threshold ${{\mathscr {D}}}=1$, see “Methods” section)

This is witnessed by the growth of maxima with *s*.

Interestingly, it was recently found in Ref.^31^ that squeezing also enhances entanglement generation if no decoherence is present, namely when the dynamics is unitary and described by the von Neumann equation (6). The present analysis thus indicates that discord (in place of entanglement) enjoys a similar property when the KTM dissipator is added to the ME [cf. Eq. (8)].

### Quantum correlations in the dissipative KTM model

As previousy discussed, the KTM model lacks a stationary state, namely there exists no solution of master equation (8) such that ${\dot{\rho }}=0$. This entails a temporal divergence of the energy of the masses. To overcome this pitfall, a dissipative version of the KTM model (henceforth referred to as DKTM model) was very recently proposed in Ref.^10^. This model produces a master equation featuring the same Hamiltonian term as Eq. (8) but a different dissipator in such a way that, differently from the KTM model, a stationary state exists (in particular preventing from energy divergence). As the original KTM model, this modified model still relies on LOCC operatios meaning that it still describes gravitational interactions mediated by a classical channel unable to create entanglement.

In the following, after briefly reviewing the DKTM model’s main features and associated master equation, we work out the ensuing equation of motion for the covariance matrix and then use it to investigate the dynamics of quantum correlations.

#### Dissipative KTM model

Like the KTM model, the DKTM model assumes that the gravitational interaction is mediated by a classical channel via local measurements and feedback. Yet, measurements of ${\hat{x}}_j$ (position of the *j*th mass) in the KTM model are now replaced by a measurements of the quadrature ${\hat{x}}_j + i \alpha {\hat{p}}_j$ with $\alpha$ a free parameter of the model that should be yet intended as small (the KTM model is retrieved for $\alpha =0$).

The resulting master equation (see Ref.^10^ for details on the derivation) reads

20 $$\begin{aligned} {\dot{\rho }}= & {} -i [{\hat{H}}+\delta {\hat{H}},\rho ] +\eta \omega \sum _{j=1}^2 \mathcal D[{\hat{X}}_j]\rho - \tfrac{i}{2}{\tilde{\alpha }} \eta \omega \sum _{j} [{\hat{X}}_j,\{{\hat{P}}_j,\rho \}] \nonumber \\&- \tfrac{1}{4} {\tilde{\alpha }}^2 \eta \omega \sum _{j} [{\hat{P}}_j,[{\hat{P}}_j,\rho ]] + \tfrac{ 1 }{2}{\tilde{\alpha }}\eta \omega \sum _{j\ne j'} [{\hat{X}}_j,[{\hat{P}}_{j'},\rho ]]\, \end{aligned}$$

with

21 $$\begin{aligned} \delta {\hat{H}}=\tfrac{1}{4} \sum _{j} {\tilde{\alpha }} \eta \omega \, ({\hat{X}}_j {\hat{P}}_j+{\hat{P}}_j{\hat{X}}_j )\,,\, \end{aligned}$$

where ${\hat{X}}_j$ and ${\hat{P}}_j$ are rescaled positions and momenta, ${\hat{H}}$ and ${\mathcal {D}}$ are the same as in Eq. (3) and where we rescaled $\alpha$ as ${\tilde{\alpha }}=m\omega \alpha$ (dimensionless).

It can be cheked that, setting $\alpha =0$, one recovers the master equation of the KTM model [cf. Eq. (7)].

#### Quantum discord

The equation of motion for the covariance matrix $\sigma$ corresponding to master equation (20), which can be worked out analogously to the KTM model, is given by Eq. (11) but with matrices *Y* and *D* now given by

22 $$\begin{aligned} Y = \left( \begin{array}{cccc} Y_{11} &{} \quad Y_{12} \\ Y_{12}&{} \quad Y_{11} \\ \end{array} \right) , \qquad \text {where}\,\,\,\,\,\,\, Y_{11} = \omega \left( \begin{array}{cccc} \frac{1}{2} {\tilde{\alpha }} \eta &{} \quad 1 \\ (\eta -1) &{} \quad \,\,-\frac{3}{2} {\tilde{\alpha }} \eta \end{array} \right) ,\,\,\,\,\, Y_{12} = \left( \begin{array}{cccc} 0 &{} \quad 0\\ -\eta \omega &{} \quad 0 \end{array} \right) , \end{aligned}$$

and

23 $$\begin{aligned} D = \left( \begin{array}{cccc} D_{11} &{} \quad D_{12} \\ D_{12}&{} \quad D_{11} \\ \end{array} \right) , \qquad \text {where}\,\,\,\,\,\,\, D_{11} = \tfrac{ \omega }{2} \left( \begin{array}{cccc} \frac{1}{2}{\tilde{\alpha }} ^2 \eta &{} \quad \quad 0\\ 0 &{} \quad \eta \omega \end{array} \right) ,\,\,\,\,\, D_{12} =\tfrac{ \omega }{2} \left( \begin{array}{cccc} 0&{} \quad \frac{1}{2}{\tilde{\alpha }} ^2 \eta \\ \frac{1}{2}{\tilde{\alpha }} ^2 \eta &{} \quad 0 \end{array} \right) . \end{aligned}$$

**Figure 5:**
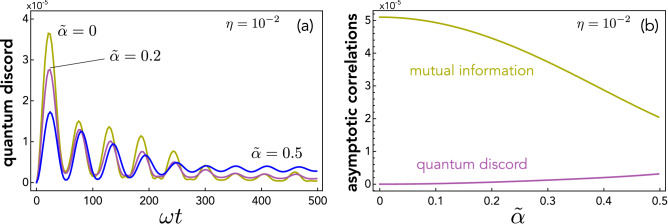
Correlations in the dissipative KTM model. (**a**): Discord ${{\mathscr {D}}}$ against time (in units of $\omega ^{-1}$) when each mass starts in a coherent state and for some representative values of parameter $\tilde{\alpha }$ (this must be small in fact by definition^10^). The case $\tilde{\alpha }=0$ (standard KTM model) is also displayed for comparison. (**b**): Asymptotic discord (purple line) and mutual information (yellow) versus $\tilde{\alpha }$. In both panels we set $\eta =10^{-2}$. Discord ${{\mathscr {D}}}$ was calculated using Eq. (27).

The typical time behaviour of quantum discord when each mass starts in a coherent state is reported in Fig. 5 for representative values of parameter $\tilde{\alpha }$, showing that discord is created even in the present model.

Somewhat similarly to the standard KTM model (cf. Fig. 3) ${{\mathscr {D}}}$ grows from zero exhibiting secondary oscillations in the transient. In contrast to the KTM model, however, ${{\mathscr {D}}}$ rapidly saturates to a small stationary value that depens on $\tilde{\alpha }$.

The occurrence of a steady value reflects the fact that the model is constructed so as to admit a stationary state. Indeed, using standard methods of linear algebra, it can be shown that the matrix equation ${\dot{\sigma }}=0$ [cf. Eq. (11)] admits only one solution $\sigma _\text {ss}$ (its analytical expression is too cumbersome to be reported here), which is the covariance matrix of the unique steady state of master equation (20). The computed discord of $\sigma _\text {ss}$ using the parameters set in Fig. 5a correctly matches the asymptotic value of ${{\mathscr {D}}}$ in each considered case. This asymptotic value slightly grows with ${\tilde{\alpha }}$ as shown in Fig. 5b, where the asymptotic mutual information is also plotted for comparison.

Overall, taking into account that $\tilde{\alpha }$ should be understood as a small parameter, we can conclude that the present model predicts that at large times classical correlations dominate over quantum ones somewhat in line with the KTM model.

## Discussion

Whether the quantum nature of the gravitational field, if any, could be witnessed by detection of quantum correlations (QCs) between two massive objects is currently a hot theme. Within this general framework, some theories have been put forward which show that the gravitational interaction could be mediated by a classical channel relying on LOCC operations, hence unable to create entanglement. In this work, we asked whether such theories might still be compatible with establishment of QCs although not accompanied by entanglement (as measured by quantum discord). To this aim, we considered the Kafri Taylor Milburn (KTM) model and its associated master equation for a pair of quantum harmonic oscillators. Based on this, we predicted production of a significant amount of quantum discord, which yet eventually decays with time. Interestingly, an initial amount of local squeezing in the oscillators can greatly enhance the maximum value reached by discord in the transient, which is reminiscent of a similar property for entanglement recently shown in the case of quantum channels. Finally, we investigated the dynamics of QCs in a recently proposed dissipative extension of the KTM model, whose ensuing master equation admits a steady state (unlike the KTM model). Similarly to the KTM model, we showed that significant discord is created with a small fraction even surviving indefinitely.

We point out that, despite its usual detrimental action for entanglement generation, decoherence plays a key role in the creation of discord without entanglement. The reason is that discord can be non-zero for separable states only provided that these are mixed. In this respect, it is therefore essential for discord creation having a non-zero dissipator in the master equation in addition to the Newtonian interaction Hamiltonian.

These findings contribute to the general debate on the possible coexistence between classical gravity and quantum mechanics by showing that, even if the gravitational field were fully classical, it might still be able to establish non-classical correlations between quantum masses although of a non-entangled nature. It is worth noting that this further highlights the importance of detecting entanglement if the goal is demonstrating the quantum nature of the gravitational field, since other forms of quantum correlations can raise from classical gravitation.

## Methods

### Computation of quantum correlations

The *total* amount of correlations between the two masses is measured by the mutual information $\mathscr{I}$^12,37^. This is defined in terms of the von Neumann entropy $S(\varrho )=-\text {Tr}(\varrho \log \varrho )$ with $\varrho$ a generic quantum state (represented by a density matrix). If $\rho$ is the joint state of the two masses, mutual information ${\mathscr {I}}$ is the discrepancy between the sum of the local von Neumann entropies and the von Neumann entropy of the joint system according to

24 $$\begin{aligned} {\mathscr {I}}= S_{1}+S_{2}-S\,. \end{aligned}$$

Here, $S_{j}=-\text {Tr}(\rho _{j}\log \rho _{j})$ is the local entropy of mass *j* [whose reduced state is given by $\rho _{1(2)}=\mathrm{Tr}_{2(1)}\rho$], while $S=-\text {Tr}(\rho \log \rho )$ is the joint entropy. A basic property of mutual information is that it vanishes if and only if $\rho =\rho _{1}\otimes \rho _{2}$, meaning that the two subsystems are fully uncorrelated. As such, mutual information captures all possible correlations regardless of their classical or quantum nature.

In this work, our main focus is quantifying the amount of *quantum* correlations (QCs) which can be measured through the so called *quantum discord*^13–15^. This quantity expresses the discrepancy between ${{\mathscr {I}}}$ and another expression of the mutual information, ${{\mathscr {J}}}=S_1-S_{1|2}$ with $S_{1|2}$ the conditional entropy^12,37^ (amount of information to describe the state of subsystem 1 conditioned to a *local* measurement of 2), which provides the same result in a classical system, but different results in a quantum system. Discord turns out to be evaluated as

25 $$\begin{aligned} {\mathscr {D}}=S_{1}-S+\underset{{\hat{M}}_{1k}}{{\mathrm{min}}}\,\,\sum _k p_k S(\rho _{2|k})\,. \end{aligned}$$

Here, the minimization is over all possible quantum measurements $\{{\hat{M}}_{1k}\}$ performed on mass $j=1$. One such measurement with outcome *k* collapses the joint system onto $\rho _{2|k}=({\hat{M}}_{1k} \rho {\hat{M}}_{1k} )/p_k$ with probability $p_k$ [note that discord is generally asymmetric, i.e., swapping indexes 1 and 2 in (25) generally changes the result. Yet, we will be interested throughout in dynamics and initial states which are invariant under the swap $1\leftrightarrow 2$].

It is worth noting that this discrepancy arises from *non-local* correlations between the two subsytems, namely a genuinely non-classical feature: discord (25) is accordingly interpreted as a measure of the total amount of QCs.

Most importantly, especially for our purposes here, while non-zero entanglement entails non-zero discord ${{\mathscr {D}}}$ the converse does not hold. Indeed, there exist *mixed* states which are separable (zero entanglement) but such that ${{\mathscr {D}}}\ne 0$ [for instance, given two spin-1/2 particles each with basis $\{|{\uparrow }\rangle$, $|{\downarrow }\rangle \}$, the state $\rho =1/2 |\uparrow \rangle _{1}\!\langle \uparrow |\otimes |\uparrow \rangle _{2}\!\langle \uparrow |+1/2 |+\rangle _{1}\!\langle +|\otimes |\downarrow \rangle _{2}\!\langle \downarrow |$, where $|+\rangle =1/\sqrt{2}\,(|\uparrow \rangle +|\downarrow \rangle )$, is separable but ${{\mathscr {D}}}\ne 0$. This is a consequence of the lack of distinguishability of states in quantum mechanics (in this case $|\uparrow \rangle$ and $|+\rangle$), which in turn follows from the superposition principle].

For Gaussian states, which are the only ones entering our analysis, the minimization in (25) can be restricted to Gaussian measurements^35^, which produces a closed analytical expression of ${{\mathscr {D}}}$ (see below) as an explicit function of the $\sigma$’s entries^27,28^ (so called Gaussian discord).

A property of Gaussian discord is that states such that $\mathscr D>1$ are entangled^28^, which somehow reflects the aforementioned ability of discord to detect QCs more general than entanglement. A consequence of this is that for any classical channel (namely unable to create entanglement as in the KTM model) if ${{\mathscr {D}}}(0)<1$ then discord must remain below the threshold ${{\mathscr {D}}}=1$ at any time, that is ${{\mathscr {D}}}(t)<1$.

### Calculation of Gaussian quantum discord

Due to the demanding minimization over all possible measurements, quantum discord as defined in Eq. (25) cannot be calculated explicitly in closed form for an arbitrary state, not even in the simplest case of a qubit (two-level system). However, for a pair of quantum harmonic oscillators which are in a two-mode Gaussian state, it can be shown^28^ that it is not restrictive limiting the minimization to *Gaussian* measurements, which yields a closed analytical formula. To give this, we first note that a generic $4\times 4$ covariance matrix has the form

26 $$\begin{aligned} \sigma = \left( \begin{array}{cc} A_1 &{} \quad A_3 \\ A_3^{\text {T}} &{} \quad A_2 \\ \end{array} \right) \,, \end{aligned}$$

where each $A_j$ is a $2\times 2$ matrix. Defining now $I_j=\det A_j$ (for $j=1,2,3$), $I_4=\det \sigma$ and $\Delta =I_1+I_2+2 I_3$, $2\nu ^2_\pm = \Delta \pm \sqrt{\Delta ^2\pm 4 I_4}$, discord (25) takes the closed form

27 $$\begin{aligned} {\mathscr {D}} = f\left( I_2\right) -f\left( \nu _-\right) -f\left( \nu _+\right) +\delta \end{aligned}$$

where

28 $$\begin{aligned} \delta = {\left\{ \begin{array}{ll} \frac{2I_3^2+(I_2-1)(I_4-I_1)+2|I_3|\sqrt{I_3^2+(I_2-1)(I_4-I_1)}}{(I_2-1)^2} \qquad \text {if } (I_4-I_1I_2)^2<(I_2+1)(I_3+I_4)I_3^2 \\ \frac{I_2I_1-I_3^2+I_4-\sqrt{I_3^4+(I_4-I_2I_1)^2-2I_3^2(I_4+I_2I_1)}}{I_2^2} \qquad \text {otherwise} \end{array}\right. } \end{aligned}$$

Equation (27) holds for measurements made on subsystem 2 (for measurements on 1 indexes 1 and 2 must be swapped).

## Data Availability

The datasets used and/or analysed during the current study available from the corresponding author on reasonable request.
